# Investigation of the Effect of Rice Wine on the Metabolites of the Main Components of Herbal Medicine in Rat Urine by Ultrahigh-Performance Liquid Chromatography-Quadrupole/Time-of-Flight Mass Spectrometry: A Case Study on *Cornus officinalis*

**DOI:** 10.1155/2013/306712

**Published:** 2013-04-29

**Authors:** Gang Cao, Hao Cai, Xianke Yue, Sicong Tu, Baochang Cai, Zhiwei Xu

**Affiliations:** ^1^Engineering Center of State Ministry of Education for Standardization of Chinese Medicine Processing, Nanjing University of Chinese Medicine, Nanjing 210023, China; ^2^Research Center of TCM Processing Technology, Zhejiang Chinese Medical University, Hangzhou 310053, China; ^3^National First-Class Key Discipline for Science of Chinese Materia Medica, Nanjing University of Chinese Medicine, Nanjing 210023, China; ^4^Faculty of Medicine, University of New South Wales, Sydney, NSW 2031, Australia; ^5^Nanjing Haichang Chinese Medicine Group Corporation, Nanjing 210061, China

## Abstract

Ultrahigh-performance liquid chromatography-quadrupole/time-of-flight mass spectrometry (UPLC-QTOF/MS) was developed for rapid and sensitive analysis of the effect of rice wine on the metabolites of the main components of herbal medicine in rat urine. Using *Cornus officinalis* as a model of herbal medicine, the metabolite profiles of crude and processed (steaming the crude drug presteeped in rice wine) *Cornus officinalis* extracts in rat urine were investigated. The metabolites of *Cornus officinalis* were identified by using dynamic adjustment of the fragmentor voltage to produce structure-relevant fragment ions. In this work, we identified the parent compounds and metabolites of crude and processed *Cornus officinalis* in rats. In total, three parent compounds and seventeen new metabolites of *Cornus officinalis* were found in rats. The contents of the parent compounds and metabolites *in vivo* varied significantly after intragastric (i.g.) administration of aqueous extracts of crude and processed *Cornus officinalis*. Data from this study suggests that UPLC-QTOF/MS could be used as a potential tool for uncovering the effects of excipients found in the metabolites of the main components of herbal medicine, *in vivo*, to predict and discover the processing mechanisms of herbal medicine.

## 1. **Introduction**

The processing of Chinese materia medica is based on traditional Chinese medicine (TCM) and has undergone continual development and refinement for thousands of years. Traditional processing and treatment of TCM utilize a range of decoction pieces, with the aim of altering the nature of the medicine to accommodate different clinical dispensings and preparation requirements [1, 2]. During processing, fire and wine are utilized in heat and excipient treatments in many herbs, respectively [3]. The processing of Chinese materia medica with excipients has a long history and the efficacy of treatment can be enhanced by using a combination of excipient treatments. There is a close relationship between the nature of Chinese materia medica and excipient treatments, through the collaboration and regulatory roles of excipients. As a result, the inherent property of such medicine can be improved through these processes to improve its treatment efficacy, which is often achieved by heating the medicine and excipient together. Solid excipients are mainly used to suppress the toxicity, while liquid excipients are used to enhance the treatment efficacy [4]. 

In processing TCM, wine is typically used as a gas component. It is believed that wine has the function of promoting blood circulation for removing obstruction in collaterals, strengthening the spleen and stomach, and removing foul odors [5, 6]. Through modern medicine, these therapeutic efficacies have been demonstrated as alcohol can dilate blood vessels to enhance cerebral blood flow, stimulate the central nervous system, and improve circulatory of the digestive system [7]. For processing TCM, rice wine is one of the most commonly used liquid excipients. Typically, rice wine is used for steeping, boiling, and steaming herbs, or making various pills and medicated wine [8]. Rice wine contains large amounts of proteins, carbohydrates, vitamins, organic acids, esters, and minerals [9]. During the saccharification and fermentation process of rice wine production, starch and protein are degraded into oligosaccharides, peptides, amino acids, and other ingredients that are readily absorbed by the human body, thus making rice wine a highly nutritious supplement. In addition, rice wine serves as a good organic solvent and has good solubility for a variety of chemical compounds and good tissue penetration capability. When the drugs are heated with wine, rice wine can change the property of medicine, lead the medicine uplink, and change the ups and downs of the Chinese medicine. It can also enhance drug efficacy and reduce foul taste and corrosion. Therefore, the processing of Chinese materia medica using rice wine offers many clinical benefits.

Dried sarcocarp of *Cornus officinalis *Sieb. et Zucc (Cornaceae) is a herbal medicine widely used in TCM for medical, food sanitation, and cosmetic purposes [10]. Clinically, it is used both in its crude herbal form and as a processed product [11]. While potent unprocessed, pharmaceutical processing may reduce toxicity or side efficacies, potentiate the beneficial effects, change the pharmacological properties, preserve active constituents, facilitate administration, improve flavor or eliminate unpleasant taste, and increase purity of herbal medicine [12, 13]. Extensive phytochemical and pharmacological studies of *Cornus officinalis* have isolated and characterized a total of 10 iridoids, most of which have been proven to have bioactive properties for the prevention and treatment of diabetic nephropathy and kidney deficiency, anti-inflammation, antivirus and antioxidation [14–16]. *Cornus officinalis*, after being stewed with yellow rice wine, has a stronger efficacy on nourishing kidneys, astringing semen, and reducing urination. It has been used diffusely for curing dizziness, coldness, pain in the waist, frequent micturition, enuresis, impotence, and prospermia [17]. Furthermore, *Cornus officinals* warmly dredges up the dint of wine and reduces its acidity. 

Within the literature, the majority of studies examining drug metabolism have been based on studies in which the liver was used as the experimental organ [18]. It is now clear that kidney also plays a major role in drug metabolism, as drug compounds and metabolites are excreted into the urine. Although most pharmacokinetic parameters of iridoids, such as morroniside, loganin, and sweroside, in *Cornus officinals* have been investigated [19–21], there is no report of renal excretion of active constituents and metabolites of main components in crude *Cornus officinalis* and its processed form. The aim of our study was to examine whether the absorption and metabolism of active components in *Cornus officinalis* through renal excretion were altered after processing using rice wine. In the present study, we employed a new and rapid ultrahigh-performance liquid chromatography-quadrupole/time-of-flight mass spectrometry (UPLC-QTOF/MS) method to investigate the effect of rice wine on the metabolites of the main components of *Cornus officinalis* in rat urine.

## 2. **Experimental**

### 2.1. Materials, Chemicals, and Reagents

Crude *Cornus officinalis* was acquired from Henan suppliers and its processed form was treated according to the Chinese Pharmacopoeia (2010 eds.). HPLC grade acetonitrile and methanol were obtained from Merck (Darmstadt, Germany) and Fisher Scientific Corporation (Loughborough, UK), respectively. Deionized water was purified using the Milli-Q system (Millipore, Bedford, MA, USA) and HPLC grade formic acid was purchased from Honeywell Company (Morristown, NJ, USA). Loganin was purchased from the National Institute for the Control of Pharmaceutical and Biological Products (Beijing, China). Morroniside and sweroside were obtained from Shanghai Shangyi Biotechnology Co. Ltd. (Shanghai, China). HPLC analysis indicated that the purities of all reference compounds were greater than 98%. All remaining chemicals were of analytical grade and commercially available.

### 2.2. Instrumentation and UPLC-QTOF/MS Conditions

Chromatography was performed using an ACQUITY C_18_ BEH column (150 mm × 2.1 mm i.d., 1.7 *μ*m) and CQUITYUPLC system (Waters Corp., Milford, MA, USA). The column was maintained at 40°C with a gradient elution of 0.1% formic acid in acetonitrile (solvent A) and 0.1% formic acid in water (solvent B) at 0–4.5 min (1–8.4% A), 4.5–8 min (8.4–9% A), 8–16 min (9–75% A), and 16-17 min (75–99% A). The flow rate was 0.45 mL/min, and 5 *μ*L aliquot of each sample was injected into the column. The eluent was then introduced to the mass spectrometer directly, that is, without a split. 

The eluent was introduced into the synapt high-definitionmass spectrometer (Waters Corp., Milford, MA, USA) analysis. The optimal conditions were as follows: capillary voltage of 2.5 kV, sampling cone voltage of 20 V, cone gas flow of 10 L/h, and desolvation gas flow of 700 L/h. The source and desolvation gas temperature were kept at 110 and 350°C, respectively. The data were collected and analyzed using Masslynx V 4.1 and MetaboLynx software. The mass spectrometric data were collected in full-scan mode; the *m/z* was from 100 to 1000 in positive and negative ions.

### 2.3. Preparation of Sample Solutions

100 g of powdered *Cornus officinalis* and its processed samples were soaked in 200 mL of water for 2 h at room temperature and thereafter refluxed for 2 h, respectively. The filtrate was collected and the residues were then refluxed twice in 1000 mL of water for 1.5 h. The three filtrates were combined and evaporated to the final volume of 100 mL under reduced pressure at a temperature not exceeding 60°C.

### 2.4. Animals, Drug Administration, Biological Sample Collection, and Preparation

Fifteen male adult Sprague-Dawley rats weighing approximately 300 g were obtained from the Laboratory Animal Center of Zhejiang Academy of Medical Sciences (Zhejiang, China). Animals were acclimatized for at least 5 days with alternating 12 h dark/light cycles in a climate-controlled room with the temperature maintained at 22 ± 1°C, and a relative humidity of 60 ± 10%. Water and standard laboratory food were available ad libitum. All experiments were performed according to the guidelines for the care and use of animals as established by Zhe Jiang University.

The rats were equally divided into three groups (group A: crude *Cornus officinalis* group, *n* = 5; group B: processed *Cornus officinalis* group, *n* = 5; group C: control group, *n* = 5) and housed individually in metabolic cages for the collection of urine samples. The rats were fed with standard laboratory food as well as water, ad libitum, and acclimatized to the facilities for 1 week prior to the start of experiments. The animals were fasted overnight with free access to water before the test. Crude *Cornus officinalis* and its processed extracts were administered to each rat in groups A and B, respectively, by traditional oral gavage at a dose of 2 mL. Crude *Cornus officinalis* and its processed extracts were administered once daily for 1 week, while the equivalent volume of distilled water was orally administered to each rat in the control group (group C). Urine samples were collected after 1 week following administration. The volume of each sample was accurately measured and stored at −80°C for preservation.

For analysis preparation, urine samples (200 *μ*L) were transferred to a 1.0 mL Eppendorf tube and acetonitrile (600 *μ*L) was added. This mixture was vortex-mixed for 2 min and centrifuged at 4,000 rpm for 5 min. The supernatant was separated out and blowed dry with nitrogen at 40°C. The residue was then reconstituted in 100 *μ*L acetonitrile and mixed to make final testing samples. A 5 *μ*L aliquot of the final testing samples was injected into the UPLC-QTOF/MS system for analysis after centrifugation at 15,000 ×g for 15 min.

### 2.5. Data Processing

The mass data analysis was carried out using MetaboLynx and Masslynx V 4.1 (Waters Corp., Milford, MA, USA) for *in vivo* metabolite identification. The data was processed and mass full-scan raw data were collected through MSE acquisition. The UPLC-QTOF/MS data was detected and noise reduced in both the UPLC and MS domains, such that only true analytical peaks were further processed by the software (e.g., residual noise spikes were rejected).

## 3. Results and Discussion

### 3.1. UPLC-QTOF/MS Identification of the Main Active Components in Rat Urine

**Figure 1:**
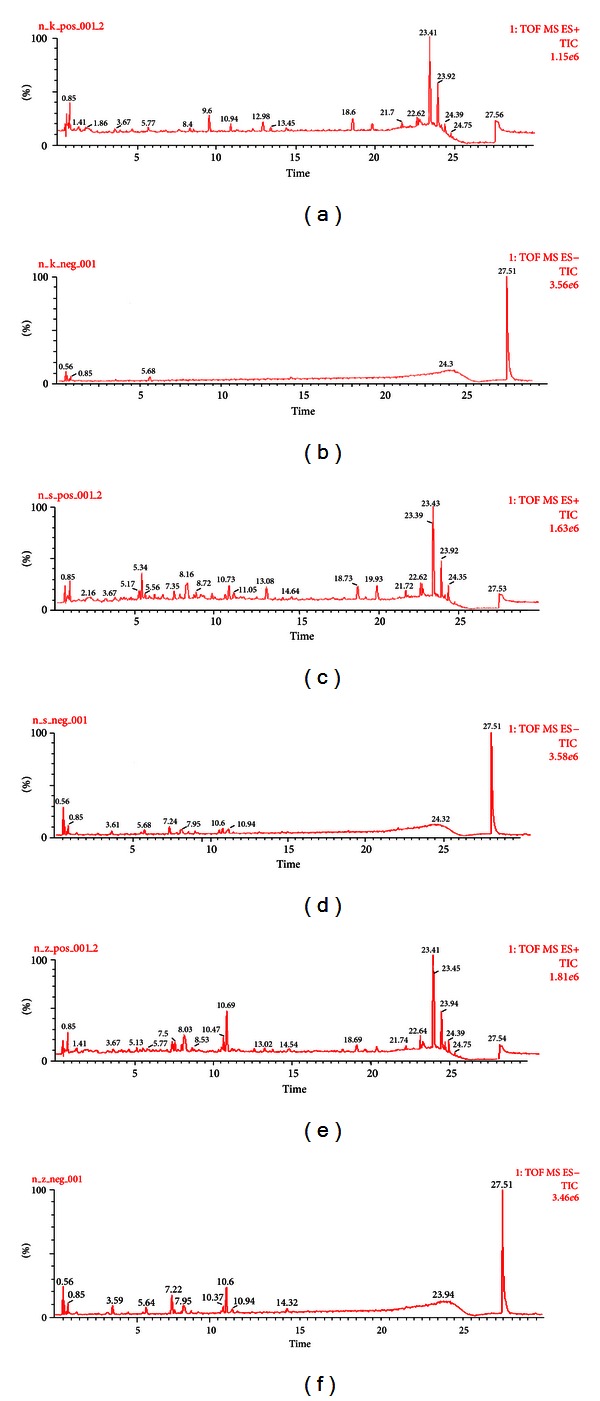
Total ion chromatograms of rat urine samples in positive and negative ion modes: (a) blank rat urine in positive mode; (b) blank rat urine in negative mode; (c) rat urine collected after administration of crude *Cornus officinalis* in positive mode; (d) rat urine collected after administration of crude *Cornus officinalis* in negative mode; (e) rat urine collected after administration of processed *Cornus officinalis* in positive mode; and (f) rat urine collected after administration of processed *Cornus officinalis* in negative mode.

Iridoid glycosides and their metabolites are main components and active compounds in *Cornus officinalis*. In the current experiment, we expected that in addition to the major iridoid glycosides in *Cornus officinalis* extract, several metabolites would be detected in rat urine. From our results, we identified three iridoid glycosides in crude *Cornus officinalis *and its processed extracts, including morroniside, loganin, and sweroside, by comparing their retention times and MS data with established standards. The ion chromatograms of three iridoid glycosides and their metabolites are presented in Figure 1, and the monitored ions of each compound are listed in Table 1. The identification of each compound is outlined below.

### 3.2. Identification of Parent Compounds and Related Metabolites

#### 3.2.1. Analysis of Parent Compound 1 and its Metabolites

Compound 1 showed an [M+COOH]^−^ ion at *m/z* 451.1456. The corresponding fragment ions, including *m/z* 101.0273 [M−C_13_H_20_O_8_]^−^, *m/z* 123.0373 [M−C_10_H_18_O_9_]^−^, *m/z* 141.0547 [M−C_10_H_16_O_8_]^−^, *m/z* 155.0334 [M−C_10_H_18_O_7_]^−^, *m/z* 179 [M−C_11_H_14_O_5_]^−^, *m/z* 243.0825 [M−C_6_H_10_O_5_]^−^, and *m/z* 405.1390 [M−H]^−^, were also identified in MS spectra of high-collision energy scan. Compound 1 was identified as morroniside through comparison with standard.

**Figure 2:**
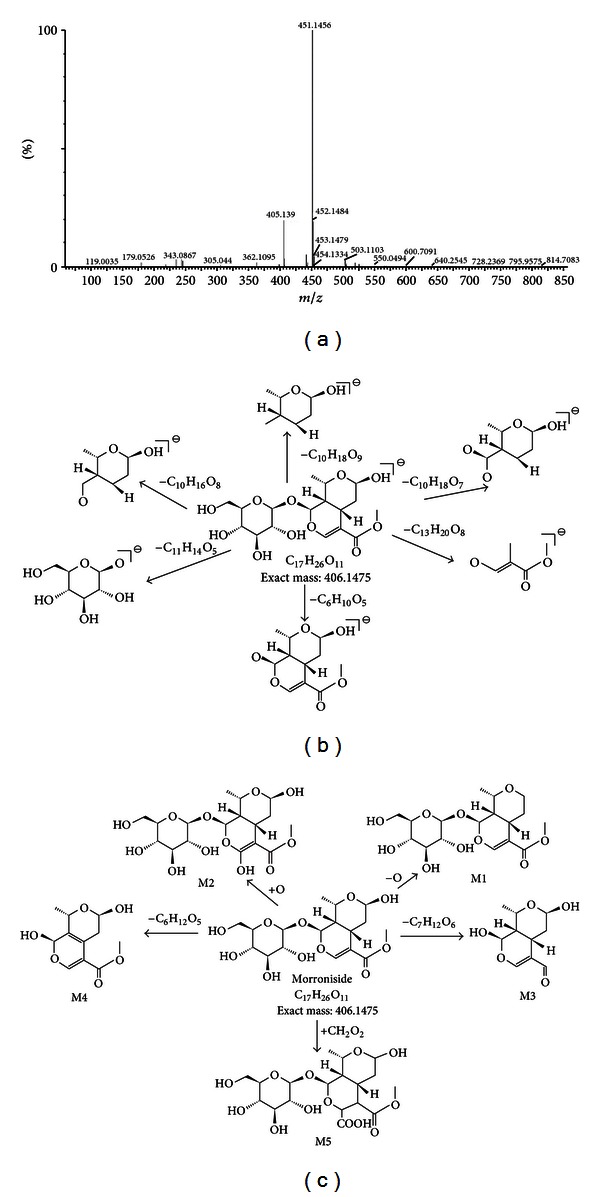
Proposed fragmentation pathways of morroniside and its metabolites from analysis of the rat urine samples: (a) accurate MS spectra of morroniside; (b) proposed fragmentation pathway of morroniside; and (c) proposed metabolic pathway of morroniside.

Five metabolites of morroniside were detected from the rat urine using MS^E^ approach and MetaboLynx, with most of the constituents [M−H]^−^ being observed in the (−) ESI-MS spectra from MS^E^ data. Moreover, the characteristic fragment peaks of the parent compound related to the metabolite were observed. The high-collision energy scan fragment ions of M1-1 at *m/z* 390.1526 [M−O]^−^, M1-2 at *m/z* 422.1195 [M + O]^−^, M1-3 at *m/z* 214.1053 [M−C_7_H_12_O_6_]^−^, M1-4 at *m/z* 225.061 [M−C_6_H_12_O_6_]^−^ (loss of a glucose group), and M1-5 at *m/z* 425.159 [M+COOH_2_]^−^ were observed. After being processed with the mass defect filter, their possible metabolites were detected in combination the with related literature data or METLIN′s metabolite mass spectral database, shown in Table 1. The pathways of morroniside and metabolites are shown in Figure 2. 

#### 3.2.2. Identification of Parent Compound 2 and Its Metabolites

Compound 2 showed an [M+COOH]^−^ ion at *m/z* 435.1512 at 30 V. It also yielded product ions, including *m/z* 101.0143 [M−C_13_H_20_O_7_]^−^, *m/z* 127 [M−C_11_H_18_O_7_]^−^, and *m/z* 227.0905 [M−C_6_H_10_O_5_]^−^. Compound 2 was identified as loganin by comparison with standard.

**Figure 3:**
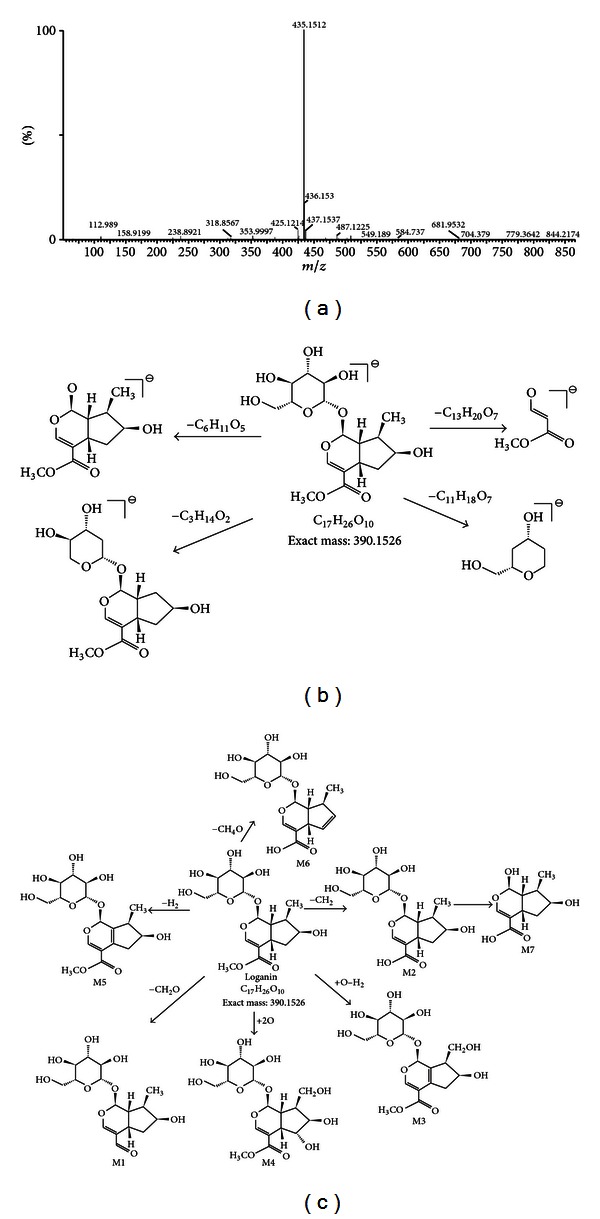
Proposed fragmentation pathways of loganin and its metabolites from analysis of the rat urine samples: (a) accurate MS spectra of loganin; (b) proposed fragmentation pathway of loganin; and (c) proposed metabolic pathway of loganin.

 Seven metabolites of morroniside were identified in the rat urine according to their retention time qualities, and MS/MS fragment ions. The high-collision energy scan fragment ions of M2-1 at *m/z* 360.1420 [M−OCH_2_]^−^, M2-2 at *m/z* 376.1363 [M−CH_2_]^−^, M2-3 at *m/z* 404.1313 [M+O−H_2_]^−,^ M2-4 at *m/z* 422.1233 [M+O_2_]^−^, M2-5 at *m/z* 388.1369 [M−H_2_]^−^, M2-6 at *m/z* 358.1264 [M−CH_4_O]^−^, and M2-7 at *m/z* 452.1590 [M−CH_2_−C_6_H_10_O_5_]^−^ were observed. The pathways of loganin and metabolites are shown in Figure 3.

#### 3.2.3. Analysis of Parent Compound 3 and Its Metabolites

Compound 3 gave an [M+Na]^+^ ion at *m/z* 381.1119 with a fragmentor voltage of 25 V. It also yielded product ions at *m/z* 127.0376 [M−C_10_H_16_O_6_]^+^ and *m/z* 197.0802 [M−C_6_H_10_O_5_]^+^. Compound 3 was identified as sweroside by comparison with standard.

**Figure 4:**
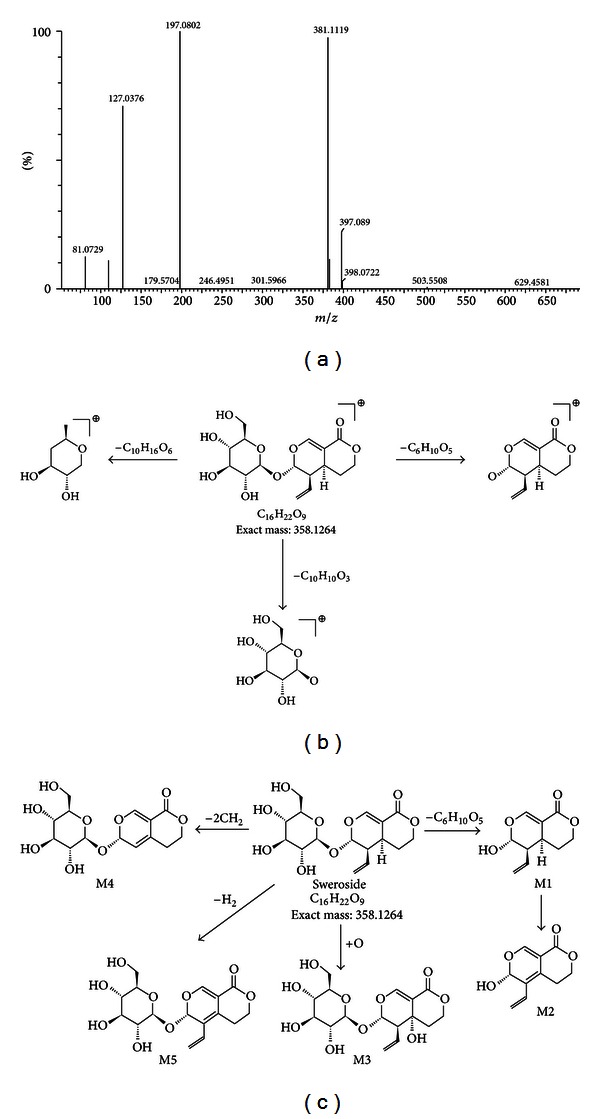
Proposed fragmentation pathways of sweroside and its metabolites from analysis of the rat urine samples: (a) accurate MS spectra of sweroside; (b) proposed fragmentation pathway of sweroside; and (c) proposed metabolic pathway of sweroside.

 Five metabolites of sweroside were detected from the rat urine using MetaboLynx software. The high-collision energy scan fragment ions of M3-1 at *m/z* 196.0736 [M−C_6_H_10_O_5_]^+^, M3-2 at *m/z* 194.0579 [M−C_6_H_10_O_5_−H_2_]^+^, M3-3 at *m/z* 374.0818 [M+O]^+^, M3-4 at *m/z* 330.0951 [M−CH_2_−CH_2_]^+^, and M3-5 at *m/z* 356.1107 [M−H_2_]^+^ were observed. The pathways of sweroside and metabolites are shown in Figure 4.

### 3.3. The Influence of Rice Wine on the Contents of Main Compounds and Their Metabolites

**Figure 5:**
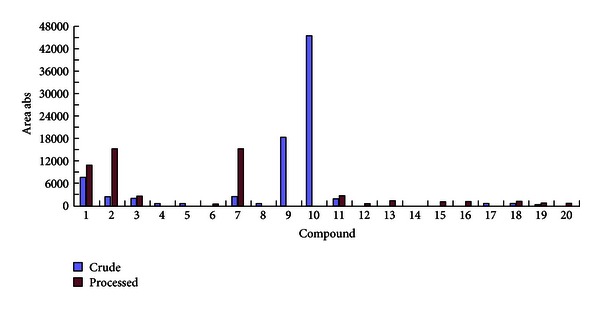
Parent compounds and metabolites detected in rat urines after oral administration of crude and processed* cornus officinalis*, respectively.

Three parent compounds and seventeen metabolites were identified in the rat urine by UPLC-QTOF/MS after i.g. administration of aqueous extracts of crude *Cornus officinalis* and its processed extracts. In crude *Cornus officinalis* samples, thirteen metabolites were detected, but metabolites including M1-5, M2-5, M2-6, M2-7, M3-1, M3-5, and one parent compound (sweroside) were not found in the rat urine. It is possible that sweroside may have been transformed into its metabolites. For processed *Cornus officinalis* samples, six metabolites, including M1-3, M1-4, M2-1, M2-2, M2-3, and M3-2, were not detected in the rat urine. The peak intensities of parent compounds and metabolites in both crude and processed* Cornus officinalis* varied significantly. Moreover, the contents of compounds were dramatically decreased in *Cornus officinalis* after processing by rice wine. The results are shown in Figure 5.

## 4. **Conclusion**

In this work, UPLC-QTOF/MS was used to investigate the excretion of extracts of crude and processed *Cornus officinalis* in rat urine. Three parent compounds and seventeen metabolites were identified, demonstrating the analytical potential of this method for metabolism studies. Our study highlights the importance of UPLC-QTOF/MS as a potential tool for uncovering the effect of rice wine on metabolites of the main components of herbal medicine, *in vivo*, to predict and discover processing mechanisms of herbal medicine. It can therefore be used for studies of excipient treatment in processing of herbal medicine. 

## Figures and Tables

**Table 1:** The mass data of metabolites and parent components acquired using UPLC-QTOF/MS.

No.	Compound	*T*_*R*_ (min)	Molecular formula	Molecular weight	QTOF/MS	Mass accuracy (ppm)
1	Morroniside	8.08	C_17_H_26_O_11_	406.1473	[M+COOH]^−^	0
2	M1-1	10.73	C_17_H_26_O_10_	390.1526	[M−O]^−^	0.6
3	M1-2	8.08	C_17_H_26_O_12_	422.1195	[M+O]^−^	0.5
4	M1-3	8.72	C_10_H_14_O_5_	214.1053	[M−C_7_H_12_O_6_]^−^	0.4
5	M1-4	5.53	C_11_H_14_O_5_	225.061	[M−C_6_H_12_O_6_]^−^	0.3
6	M1-5	23.9	C_18_H_28_O_13_	452.159	[M+COOH_2_]^−^	0.4
7	Loganin	10.60	C_17_H_26_O_10_	390.1520	[M+COOH]^−^	0
8	M2-1	7.5	C_16_H_24_O_9_	360.1420	[M−OCH_2_]^−^	0.6
9	M2-2	6.28	C_16_H_24_O_10_	376.1363	[M−CH_2_]^−^	0.2
10	M2-3	7.42	C_17_H_24_O_11_	404.1313	[M+O–H_2_]^−^	0.3
11	M2-4	8.05	C_17_H_26_O_12_	422.1233	[M+O_2_]^−^	0.5
12	M2-5	7.5	C_17_H_24_O_10_	388.1369	[M−H_2_]^−^	0.3
13	M2-6	10.47	C_16_H_22_O_9_	358.1264	[M−CH_4_O]^−^	0.1
14	M2-7	23.96	C_11_H_14_O_7_	452.1590	[M−CH_2_–C_6_H_10_O_5_]^−^	0.4
15	Sweroside	10.47	C_16_H_22_O_9_	358.1256	[M+Na]^+^	0
16	M3-1	10.48	C_10_H_12_O_4_	196.0736	[M−C_6_H_10_O_5_]^+^	0.6
17	M3-2	8.08	C_10_H_10_O_4_	194.0579	[M−C_6_H_10_O_5_–H_2_]^+^	0.2
18	M3-3	7.33	C_16_H_22_O_10_	374.0818	[M+O]^+^	0.5
19	M3-4	2.65	C_14_H_18_O_9_	330.0951	[M−CH_2_–CH_2_]^+^	0.3
20	M3-5	6.35	C_16_H_20_O_9_	356.1107	[M−H_2_]^+^	0.4
